# The inositol hexakisphosphate kinases IP6K1 and -2 regulate human cellular phosphate homeostasis, including XPR1-mediated phosphate export

**DOI:** 10.1074/jbc.RA119.007848

**Published:** 2019-06-11

**Authors:** Miranda S. Wilson, Henning J. Jessen, Adolfo Saiardi

**Affiliations:** ‡ MRC Laboratory for Molecular Cell Biology, University College London, London, United Kingdom; § Institute of Organic Chemistry, Albert-Ludwigs-University Freiburg, Freiburg, Germany

**Keywords:** inositol phosphate, cell signaling, ATP, metabolic regulation, mammal, inositol hexakisphosphate kinase (IP6K), inositol pyrophosphate, IP7, phosphate homeostasis, SPX domain

## Abstract

Phosphate's central role in most biochemical reactions in a living organism requires carefully maintained homeostasis. Although phosphate homeostasis in mammals has long been studied at the organismal level, the intracellular mechanisms controlling phosphate metabolism are not well-understood. Inositol pyrophosphates have emerged as important regulatory elements controlling yeast phosphate homeostasis. To verify whether inositol pyrophosphates also regulate mammalian cellular phosphate homeostasis, here we knocked out inositol hexakisphosphate kinase (IP6K) 1 and IP6K2 to generate human HCT116 cells devoid of any inositol pyrophosphates. Using PAGE and HPLC analysis, we observed that the IP6K1/2-knockout cells have nondetectable levels of the IP_6_-derived IP_7_ and IP_8_ and also exhibit reduced synthesis of the IP_5_-derived PP-IP_4_. Nucleotide analysis showed that the knockout cells contain increased amounts of ATP, whereas the Malachite green assay found elevated levels of free intracellular phosphate. Furthermore, [^32^P_i_] pulse labeling experiments uncovered alterations in phosphate flux, with both import and export of phosphate being decreased in the knockout cells. Functional analysis of the phosphate exporter xenotropic and polytropic retrovirus receptor 1 (XPR1) revealed that it is regulated by inositol pyrophosphates, which can bind to its SPX domain. We conclude that IP6K1 and -2 together control inositol pyrophosphate metabolism and thereby physiologically regulate phosphate export and other aspects of mammalian cellular phosphate homeostasis.

## Introduction

Phosphate homeostasis is crucial for growth and survival, because virtually all biochemical processes utilize phosphate. In mammals, a complex hormonal regulatory network involving intestines, bones, and kidneys regulates serum phosphate concentration (1). Excessive serum phosphate (hyperphosphatemia) has been linked to cardiovascular disease and increased mortality in chronic kidney disease patients, whereas hypophosphatemia can result in a form of rickets. Serum phosphate levels are maintained by FGF23-αKlotho, parathyroid hormone, and vitamin D signaling. Many cell types have been shown to transcriptionally respond to changes in extracellular phosphate concentrations, and the phosphate uptake transporter Pit1/SLC20A1 has been proposed to sense extracellular phosphate. However, how phosphate homeostasis is maintained at the cellular level remains a poorly studied area.

Analysis of yeast and plant genomes has revealed that the SPX protein domain (after SYG1, Pho81, and XPR1, Pfam: PF03105) is found in many proteins that regulate phosphate metabolism (2). The *Arabidopsis thaliana* (thale cress) genome encodes 20 proteins containing SPX domains, whereas the *Saccharomyces cerevisiae* (budding yeast) genome contains 10 SPX proteins. Four of these, Vtc2–5, form part of the VTC complex that spans the vacuolar membrane. This complex synthesizes the linear polymer inorganic polyphosphate (polyP)^2^ that functions as the main phosphate storage molecule in yeast (3). It is now known that VTC polyP synthesis requires binding of an inositol pyrophosphate, specifically the 5-diphosphoinositol pentakisphosphate (5PP-IP5; 5-IP_7_) isomer of IP_7_, to the VTC SPX domains (4, 5), explaining the observation that yeast devoid of IP_7_ lack polyP (6). IP_7_-SPX binding also promotes the interaction of two rice phosphate-regulated transcription factors, OsSPX4 (an SPX domain-containing protein) and OsPHR2. Electrophysiological measurements of the parasite *Trypanosoma brucei* SPX protein TbPho91, and its yeast homolog Pho91, suggest IP_7_ as a regulator of their phosphate transport activities (7).

Inositol pyrophosphates (PP-IPs) are *myo*-inositol–derived signaling molecules ubiquitous in eukaryotic cells (8). They are defined and distinguished from other inositol phosphates by the presence of at least one phosphoanhydride bond. The best characterized, 5-IP_7_, whose phosphoanhydride bond is at the 5-position of the *myo*-inositol carbon ring (8), is synthesized by IP6K enzymes from inositol hexakisphosphate (IP_6_; phytic acid). The IP6Ks are catalytically flexible: they can use IP_5_ as substrate to generate 5PP-IP_4_, whereas in the presence of ADP they can dephosphorylate IP_6_ to I(2,3,4,5,6)P_5_ (9). Eukaryote genomes also contain another class of kinase, namely the PPIP5Ks, able to synthesize PP-IPs. These enzymes preferentially act on 5-IP_7_, converting it to 1,5(PP)_2_-IP_4_ (called IP_8_) (10, 11). Budding yeast has one IP6K enzyme named Kcs1, whereas mammalian genomes encode three IP6K isoforms: IP6K1, IP6K2, and IP6K3. Studies using *kcs1*Δ yeast or individual IP6Ks KO mice have linked PP-IPs to a plethora of cellular activities (12), leading to the suggestion that PP-IPs act as “metabolic messenger” (13): a signaling molecule involved in the regulation of metabolic homeostasis (14). Besides reduced polyP levels (6), *kcs1*Δ yeast show reduced uptake of phosphate from the culture medium (15). This appears to be an evolutionarily conserved phenotype, because mammalian IP6K2 was initially discovered as a protein that could stimulate phosphate (P_i_) uptake into *Xenopus* oocytes, and was called PiUS (P_i_ Uptake Stimulator) before its enzymatic abilities were discovered (16–18). Furthermore, two single-nucleotide polymorphisms within the human *IP6K3* gene locus are associated with differences in serum phosphate concentration (19).

Unlike yeast or plants, mammalian genomes contain a single SPX domain-containing protein. Localized at the plasma membrane, XPR1 was originally characterized as a retroviral receptor (Xenotropic and Polytropic retrovirus Receptor 1), but is functionally a phosphate exporter (20). In the current study we aimed to investigate if and how PP-IPs regulate intracellular phosphate homeostasis in mammalian cells. Most previous work has used cells knocked out for only one IP6K at a time, generating cells with a reduction rather than complete depletion of PP-IPs levels. IP6K1 and IP6K2 have a wide and overlapping tissue distribution, whereas IP6K3 is highly expressed in skeletal muscle (21). We used CRISPR and the human colon carcinoma line HCT116 to create a cell line truly devoid of PP-IPs by disrupting both IP6K1 and IP6K2. These DKO cells showed an increased amount of ATP as well as increased intracellular free phosphate. Conversely, release as well as uptake of radioactive phosphate was reduced. Knockdown of XPR1 inhibited [^32^P_i_] release in WT cells, but had no effect in DKO cells, demonstrating that PP-IPs regulate phosphate export through XPR1.

## Results

### Generation of cells without IP_7_

**Figure 1.**
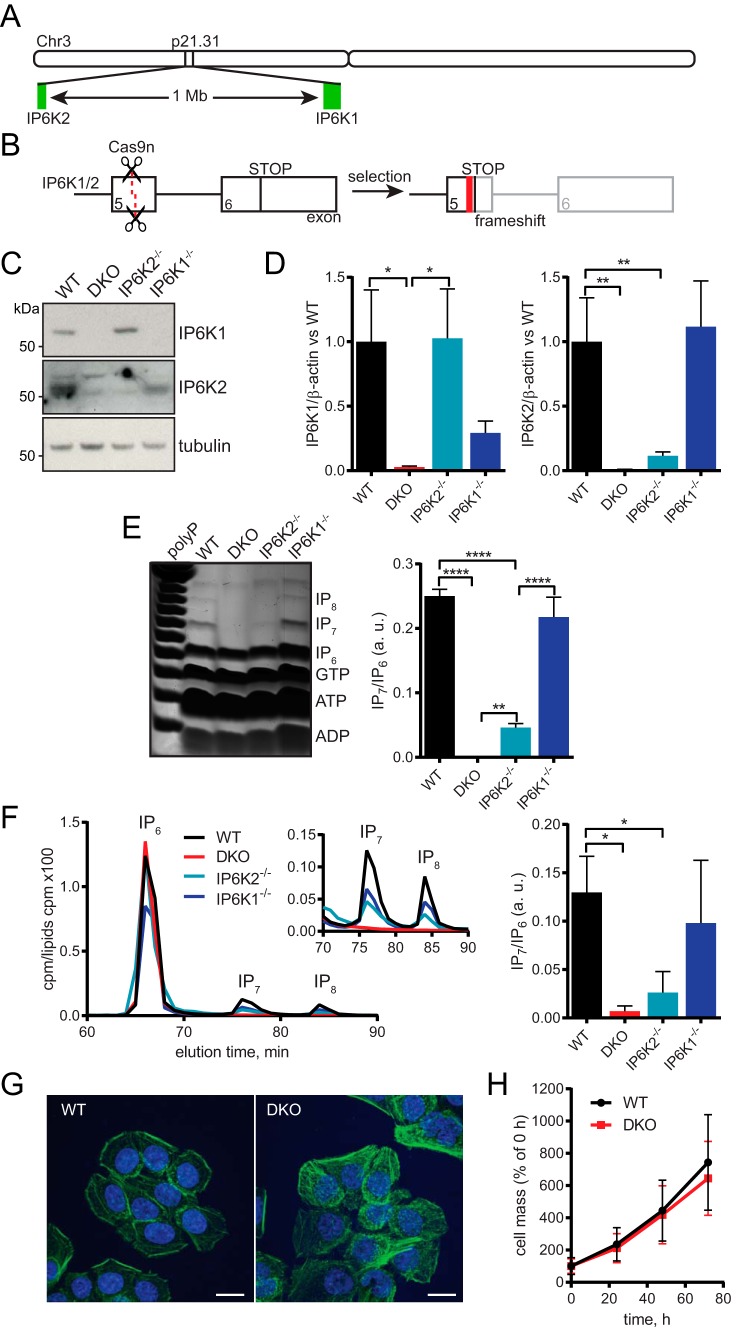
**Generation and characterization of IP6K knockout HCT116 cells.** *A,* schematic showing the localization of human *IP6K1* and *IP6K2* on chromosome 3, to scale. In mice, these genes are separated by 0.7 Mb on chromosome 9 at 9 F1 and 9 F2, respectively. *B,* schematic for creation of IP6K1/2 knockout human cells using CRISPR. Guide RNAs were designed to target exon 5 of *IP6K1* and *IP6K2*, using nickase Cas9n D10A. Successfully mutated clones contained indels that caused frameshift and premature stop codons. *C,* Western blotting for IP6K1 and IP6K2 showing their loss in KO cells. Tubulin is shown as loading control. *D,* RT-qPCR analysis of IP6K1 (*left*) and IP6K2 (*right*) mRNA transcripts in KO cells, normalized to β-actin. We were unable to detect any IP6K3 protein or mRNA. *E,* titanium dioxide-purified perchloric acid cell extracts resolved by 35% PAGE and stained with toluidine blue, with densitometric analysis (*right*). Identity of bands determined by migration compared with previous analysis of standards. Extracts equivalent to 16 mg of protein were loaded. Synthetic polyP used as a ladder. *F,* SAX-HPLC of *myo-*[^3^H]inositol–labeled cells. Cells were labeled for 5 days in inositol-free DMEM. *Inset* shows a close up of IP_7_ and IP_8_. Ratio of the IP_7_/IP_6_ peaks is shown (*right*). *G,* maximum projection images of FITC-phalloidin (*green*) stained fixed cells. Nuclei stained with Hoechst (*blue*). *Scale bars*, 15 μm. *H,* SRB cell growth assay. Data show mean ± S.D. from 11 experiments. Bar charts in *B–D* show mean ± S.D. from 3 experiments. HPLC traces in *D* and images in *E* are representative of experiments performed 3 times. *, *p* < 0.05; **, *p* < 0.01; ****, *p* < 0.0001, ANOVA with Tukey post test.

To study the role that PP-IPs play in mammalian phosphate homeostasis, we generated cells devoid of this small molecule messenger. Mammalian genomes possess three IP6K homologs. The *IP6K1* and *IP6K2* genes are located close together on the same chromosome (located at chromosome 3p21.31 in humans; in mice, chromosome 9;9 F1 and 9;9 F2 for *ip6k1* and *ip6k2*, respectively) (Fig. 1*A*). Therefore, crossing the *ip6k1*^−/−^ and *ip6k2*^−/−^ mice is unlikely to generate an *ip6k1*^−/−^*ip6k2*^−/−^ DKO mouse because of linkage. Instead, we used CRISPR to create a cell line without PP-IPs. IP6K3 is expressed mainly in muscle cells (21), and indeed the cell lines we tested, which were not muscle-derived, contained no or a very low amount of IP6K3 mRNA, whereas IP6K1 and IP6K2 were widely co-expressed (Fig. S1*A*). We chose to use the human colon carcinoma cell line HCT116 as it is near-diploid (22), expresses IP6K1 and IP6K2 only, and has easily detectable levels of IP_7_ and IP_8_ (23). We targeted *IP6K1* and *IP6K2* using guide RNAs against exon 5 of both genes to disrupt the inositol-binding motifs (Fig. 1*B*) (17).

No IP6K1 or IP6K2 protein was detectable in DKO *IP6K1*^−/−^*IP6K2*^−/−^ lines (Fig. 1*C*), which also contained significantly less IP6K1 and IP6K2 mRNA than WT cells (Fig. 1*D*). We attempted to analyze IP6K3 mRNA, in case this homolog was up-regulated to compensate, but were unable to detect any in the knockout or WT cells.

**Figure 2.**
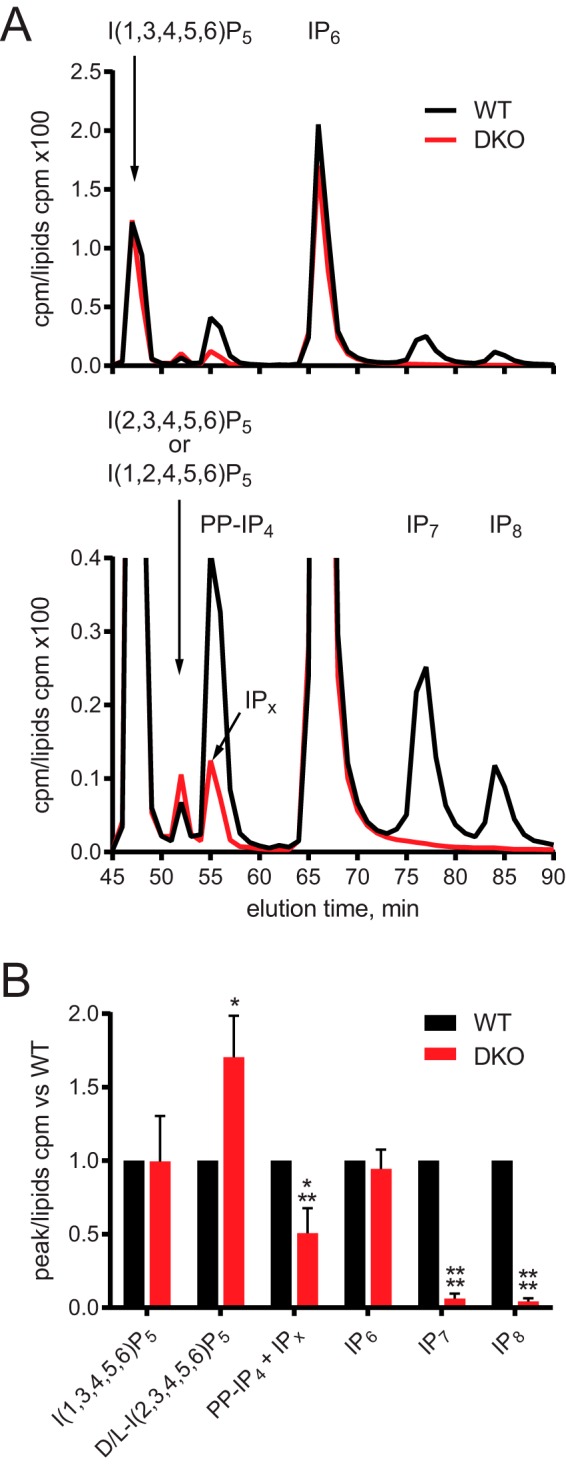
**No change in IP_6_ or I(1,3,4,5,6)P_5_ levels in DKO cells.** *A,* SAX-HPLC of *myo-*[^3^H]inositol–labeled cells. Cells were labeled for 5 days in inositol-free DMEM. Peaks of I(1,3,4,5,6)P_5_, D/l-I(2,3,4,5,6)P_5_ (the stereoisomers I(1,2,4,5,6)P_5_ and I(2,3,4,5,6)P_5_ cannot be distinguished by this HPLC method), 5PP-IP_4_, IP_6_, IP_7_, and IP_8_ were identified based on elution time compared with standards. The IP6K product 5PP-IP_4_, lost in DKO cells, coeluted with an unknown peak designated IP*_x_*. The data are shown fully (*top*) and zoomed-in to better visualize the smaller peaks (*bottom*). *B,* comparison of IPs species, normalized to WT to allow for differences in labeling between experiments. Bar chart shows mean ± S.D. from 3 or more experiments. HPLC trace in *A* is representative of an experiment performed 3 times. *, *p* < 0.05; ***, *p* < 0.001; ****, *p* < 0.0001, ANOVA with Tukey post test.

We then analyzed the effect of loss of IP6Ks on the cellular inositol phosphate profile. Similar results were obtained with both TiO_2_ beads purification PAGE analysis (Fig. 1*E*) and [^3^H]inositol labeling SAX-HPLC (Fig. 1*F*) techniques. Single IP6K knockout lines still showed detectable levels of IP_7_. In *IP6K2*^−/−^ cells, IP_7_ was significantly reduced compared with WT but was still present, whereas the *IP6K1*^−/−^ line did not have significantly less IP_7_ than WT. No IP_7_ or IP_8_ was detectable in the DKO cells, demonstrating that, while IP6K2 appears to contribute most IP_7_ in HCT116 cells, knockout of both IP6K1 and IP6K2 is necessary to completely deplete these PP-IPs. We consequently focused our attention on the DKO cell lines. These cells showed a normal level of IP_6_ and the major IP_5_ isoform I(1,3,4,5,6)P_5_ (Fig. 2). Two smaller peaks were seen eluting between I(1,3,4,5,6)P_5_ and IP_6_. One was identified by coelution with standard as the alternative IP6K product I(2,3,4,5,6)P_5_ or its indistinguishable stereoisomer I(1,2,4,5,6)P_5_ (9). As this peak increased in the DKO cells (Fig. 2*B*), *in vivo* one or both of these isomers must be synthesized through an IP6K-independent but regulated route. The second peak, strongly reduced in the DKO cells, coeluted with a standard of the IP6K product 5PP-IP_4_. The remaining signal suggests coelution of an unknown species, probably another IP_5_ isomer, that we have named IP*_x_*.

Morphologically, the DKO cells appeared similar to the WT cells (Fig. 1*G*), and there was no significant difference in cell growth (Fig. 1*H*). Treatment of mammalian cells with the phosphatase inhibitor sodium fluoride is known to increase levels of PP-IPs by inhibiting their turnover (24). Fluoride treatment of WT cells resulted in 5-IP_7_ and IP_8_ accumulation as expected (Fig. S1*B*). A band migrating slightly faster than IP_6_ is also seen, likely 5PP-IP_4_. However, the DKO cells accumulated solely the PPIP5K product 1-IP_7_, which migrates more slowly. In WT cells 1-IP_7_ represents <2% of total IP_7_ (25). Expression of the mammalian PPIP5K isoforms PPIP5K1 and PPIP5K2 was unchanged in IP6K KO cells (Fig. S1*C*).

### Mitochondria are unaffected in DKO cells

**Figure 3.**
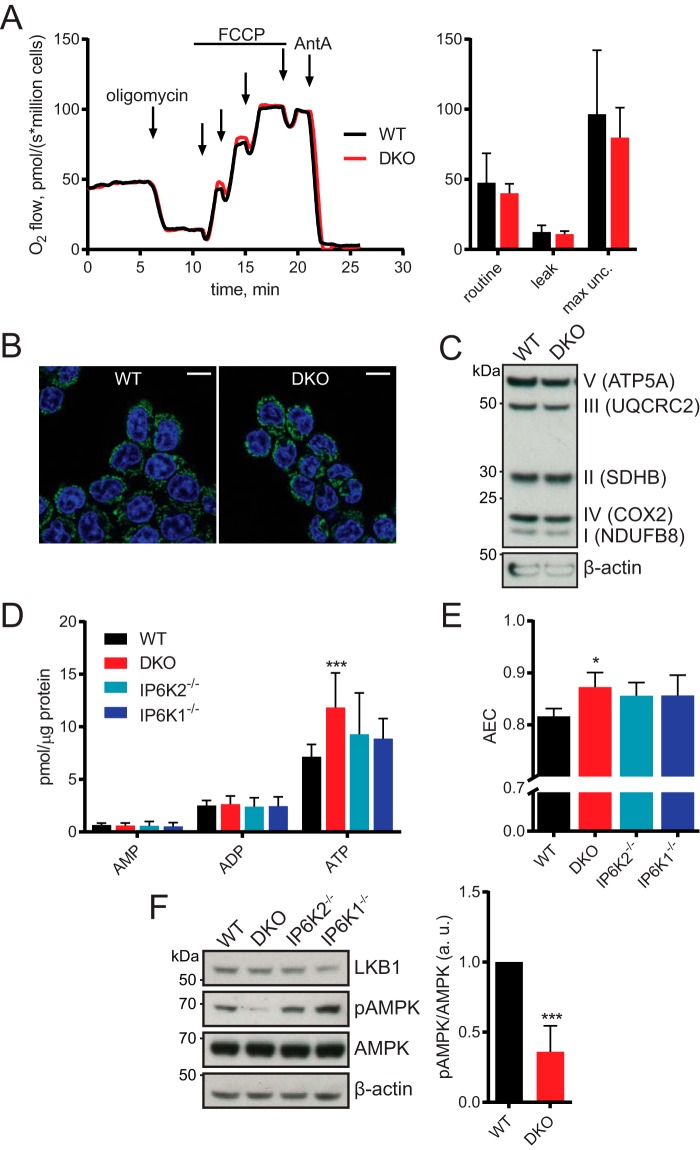
**Altered ATP levels seen in DKO cells but no difference in mitochondrial activity.** *A,* respirometric analysis of oxygen consumption over time, with addition of mitochondrial modulators. *B,* images of live cells treated with MitoTracker (shown in *green*). Nuclei were stained with Hoechst (*blue*). *Scale bars*, 15 μm. *C,* Western blotting using antibodies against members of the five electron transport chain complexes, plus actin as loading control. *D,* HPLC analysis of adenine nucleotides. *E,* adenylate energy charge ((ATP + 0.5 ADP)/(ATP + ADP + AMP)) derived from HPLC results in *D. F,* Western blotting for LKB1, total and phosphorylated AMPK (Thr^172^), and actin. Quantified by densitometry (*right*). Blot is representative of 4 experiments. ***, *p* < 0.001, *t* test. Respirometry in *A*, images in *B* and blot in *C* are representative of 3 experiments. Bar chart in *A* shows mean ± S.D. from 3 experiments. HPLC data in *D* and *E* show mean ± S.D. from 6 experiments. *, *p* < 0.05, ANOVA with Tukey post test. *max unc*., maximum uncoupled rate.

Previous work found that *kcs1*Δ yeast and *IP6K1*^−/−^ murine embryonic fibroblasts have defects in mitochondrial function (26). Surprisingly, we did not find any difference between WT and DKO cells using respirometry (Fig. 3*A*). The mitochondria looked similar following MitoTracker Deep Red staining (Fig. 3*B*), and expression of various electron transport complex subunits was unchanged (Fig. 3*C*). It is possible that the different result seen in murine embryonic fibroblasts demonstrates cell type specificity for IP_7_ function.

### ATP is increased in DKO cells

As discussed above, PP-IPs regulate phosphate storage and buffering in budding yeast by controlling polyP production. The presence, abundance, and nature of polyP in mammalian cells remains an open question, and likely will be until the relevant mammalian polyP enzymology is discovered. No true polyP null or overexpression cells are available to act as controls. We attempted to detect polyP in WT and DKO cells. Phenol extraction followed by PAGE and 4′,6-diamidino-2-phenylindole (DAPI) staining did not reveal any polyP characteristic ladders or smears (Fig. S2*A*). We then tried radioactive [^32^P_i_] labeling to maximize sensitivity. Cells were starved of phosphate for 24 h, on the assumption that if polyP was present it would be degraded during this period to provide phosphate. Phosphate was then restored, along with [^32^P_i_], for a further 24 h, which would stimulate synthesis of radiolabeled [^32^P_i_]polyP. Visualization of PAGE-resolved extracts by toluidine blue and autoradiography also failed to show any polyP-like signal (Fig. S2*B*). HCT116 cells may therefore not possess polyP; if it exists there, it is both low abundance and very short chain, requiring novel extraction procedures and detection methods more sensitive than radioactive labeling.

The absence of polyP led us to theorize that other phosphate-rich and abundant molecules, such as ATP, might work as phosphate buffers in mammalian cells. Intracellular ATP exists at millimolar concentrations (27), representing a large portion of a cell's phosphate content. Using ion-pairing HPLC we simultaneously and quantitatively measured ATP, ADP, and AMP in cell extracts (Fig. 3*D*). A 1.7-fold increase in amount of ATP was seen for DKO cells (Table S1), but there were no significant alterations in AMP or ADP level. The relative proportions of AMP, ADP, and ATP can be combined into one value by calculating the adenylate energy charge (AEC; (ATP + 0.5 ADP)/(ATP + ADP + AMP)). The DKO cells had significantly higher AEC ratio (Fig. 3*E*, Table S1). No significant alterations in ATP level or AEC were found for single *IP6K1*^−/−^ and *IP6K2*^−/−^ cell lines. Cellular sensing of energy, via sensing of AMP:ATP and ADP:ATP ratios, is performed by the AMPK (AMP-activated kinase) complex. Phosphorylation of AMPK's α subunit at Thr^172^ is required for full activity, and this is promoted by AMP binding (28). The increase in AEC in DKO cells, meaning proportionately less AMP, was reflected in a >0.6-fold reduction in AMPK phosphorylation (Fig. 3*F*).

To confirm the ATP increase following PP-IPs cellular depletion in another cell type and by using a different experimental approach, we used the yeast 5-pyrophosphatase Siw14 (29). This enzyme has no mammalian homolog. We transfected HeLa cells with a plasmid encoding either the WT humanized Myc-Siw14 or pyrophosphatase-dead C214S mutant. Overexpression of the active, but not the dead, Myc-Siw14 depleted IP_7_ and IP_8_ levels (Fig. S3*A*). As with the HCT116 DKO stable cell line, which experiences a chronic loss of PP-IPs, acute depletion by transient transfection in HeLa cells also caused a significant increase in ATP cellular level (Fig. S3*B*). In this case, levels of ADP and AMP were also higher when PP-IPs were depleted.

### Free phosphate is increased but phosphate flux is reduced in DKO cells

**Figure 4.**
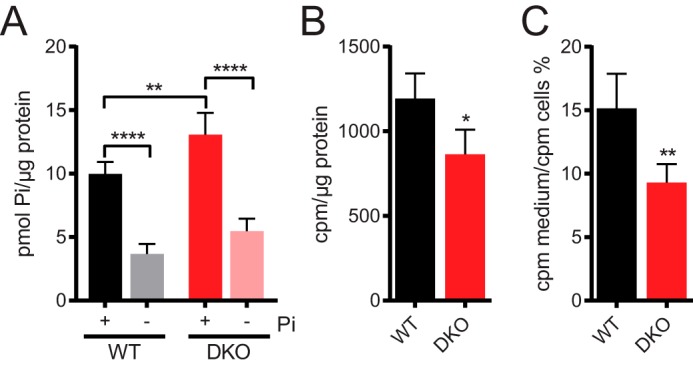
**Altered phosphate homeostasis in DKO cells.** *A,* cells were grown in 0.9 or 0 mm phosphate for 24 h before determination of intracellular free phosphate using Malachite green assay. *B,* cells were pulse labeled with [^32^P_i_] for 20 min, then intracellular [^32^P_i_] was counted using a β-counter. Results were normalized to protein concentration from untreated control wells. *C,* cells were pulse labeled with [^32^P_i_] for 20 min, washed, and incubated in unlabeled medium for 30 min. Counts for media samples were normalized by cells counts. Labeling experiments with [^32^P_i_] were performed in media containing 0.9 mm phosphate. Data in *B* and *C* show mean ± S.D. from 4 experiments. *, *p* < 0.05; **, *p* < 0.01, *t* test. Chart in *A* shows mean ± S.D. from 5 experiments. **, *p* < 0.01; ****, *p* < 0.0001, ANOVA with Tukey post test. No significance was found between 0 mm phosphate-starved WT and DKO cells. P_i_ = phosphate.

The increased adenylate pools in DKO likely reflects an increase in total cellular phosphate content. We next investigated if free phosphate levels were also affected. Indeed, DKO cells contained 1.3-fold more free phosphate compared with WT cells (WT 10.0 ± 0.9 *versus* DKO 13.1 ± 1.7 pmol of phosphate/μg of protein; Fig. 4*A*). Starving the cells of phosphate for 24 h decreased free intracellular phosphate in both lines to 0.4-fold of the phosphate-replete value; there was no significant difference in phosphate concentration in starved cells.

The absence of PP-IPs in DKO cells therefore results in an increase in both free phosphate and bound phosphate, in the form of adenylates. Such phosphate overload could negatively influence phosphate flux in and out of the cells. To test this hypothesis, the steady-state results were complemented by [^32^P_i_] pulse labeling experiments to analyze flux (Fig. 4, *B* and *C*). To maximize physiological relevance, culture medium containing the usual phosphate concentration was used, rather than phosphate-free medium. Both [^32^P_i_] uptake and release were significantly reduced in the DKO cells: 0.7-fold [^32^P_i_] was taken up, and 0.6-fold [^32^P_i_] released back into the medium, validating our hypothesis.

### Phosphate release in HCT116 cells is XPR1- and PP-IP_7_-dependent

Release of [^32^P_i_] from labeled cells into the medium is XPR1-dependent (20). This protein is the only identified mammalian phosphate exporter, but several phosphate importers (sodium-phosphate cotransporters) are known, across three classes: SLC17, SLC34, and SLC20 (1). We were only able to detect mRNA for SLC20A1/Pit1 in HCT116 cells. There was no statistically significant difference in expression of Pit1 or XPR1 (Fig. S4, *A* and *B*) between WT and DKO cells.

**Figure 5.**
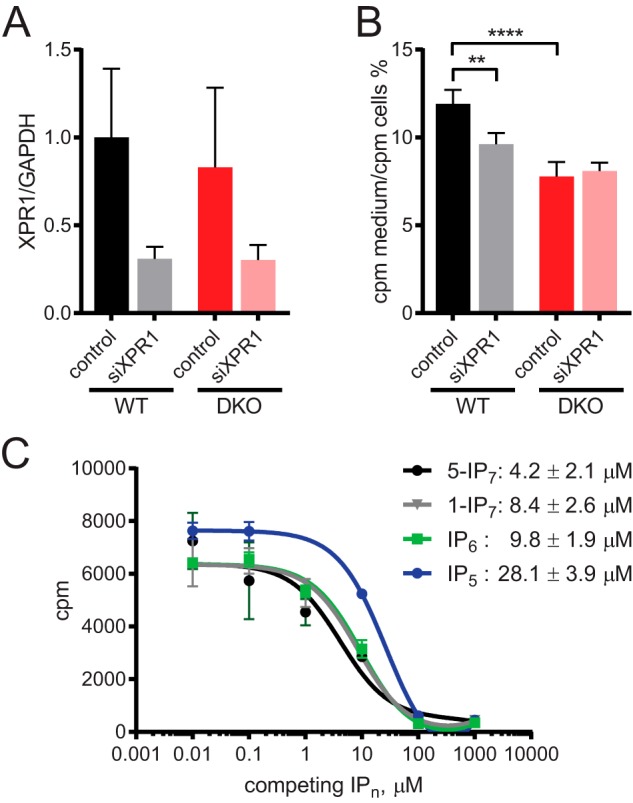
**XPR1 exports [^32^P_i_] in a PP-IPs–dependent manner.** *A,* cells were transfected for 24 h with siRNA against XPR1 or control scramble siRNA. RT-qPCR was used to confirm knockdown, using primers against GAPDH as control. *B,* release of [^32^P_i_] from pulse labeled cells 24 h post-transfection with siXPR1 or control siRNA. *C,* competition-binding assay using recombinant SPX domain of human XPR1. SPX^XPR1^ was preincubated at room temperature with [^3^H]IP_6_ for 5 min, then incubated with unlabeled inositol phosphates for 15 min. The protein was PEG-precipitated, and radioactivity was counted using a β-counter. IC_50_ values for competing with [^3^H]IP_6_ are shown. Data in *A* show mean ± S.D. from 3 experiments. Data in *B* show mean ± S.D. from 4 experiments. **, *p* < 0.01; ****, *p* < 0.0001, ANOVA with Tukey post test.

XPR1 is also the only mammalian SPX domain-containing protein. As PP-IPs are known to regulate the activity of SPX proteins from other systems, we were interested in a closer look at the reduced phosphate efflux phenotype. We knocked down XPR1 using siRNA. Commercial antibodies for XPR1 are unable to detect endogenous protein by Western blotting. Therefore, we relied on RT-qPCR to record a reduction in XPR1 mRNA (Fig. 5*A*). Transient knockdown of XPR1 significantly reduced [^32^P_i_] release 0.8-fold in WT HCT116 cells (Fig. 5*B*), again confirming its role as phosphate exporter (20). However, siXPR1 did not further affect the low [^32^P_i_] release from DKO cells. This shows that in WT cells phosphate is exported through XPR1 in an IP6K1/2-generated PP-IPs-dependent manner.

The ability of XPR1's SPX domain to bind IP_6_ has been shown by NMR (4). To investigate if PP-IPs such as IP_7_ are XPR1 ligands, we incubated purified SPX^XPR1^ with [^3^H]IP_6_ for a competition-binding assay (Fig. 5*C*). Titration of unlabeled IP_7_ reduced the radioactive counts, meaning that it was able to compete with the radioactive [^3^H]IP_6_ for binding. We observed similar IC_50_ values for competing for binding for IP_6_, 1-IP_7_, and 5-IP_7_ of ∼9.8, 8.4, and 4.2 μm, respectively, whereas I(1,3,4,5,6)P_5_ displaced IP_6_ with a lower affinity. Therefore, IP_7_ can bind directly to this SPX protein. Similar affinities for IP_6_ and IP_7_ appears to be a common characteristic of SPX domains from various proteins. However, as seen here for XPR1, studies of the yeast VTC complex also revealed functional selectivity for IP_7_ over IP_6_ (5).

## Discussion

To uncover a role for PP-IPs in mammalian phosphate regulation we knocked out IP6K1 and IP6K2 in human HCT116 cells. The DKO cells lacked any detectable IP_7_ or IP_8_. These cells contained more intracellular free phosphate and ATP than WT. As IP_7_ has been shown in other systems to regulate SPX proteins, we investigated the mammalian SPX protein, phosphate exporter XPR1. Knockdown of XPR1 reduced [^32^P_i_] cellular release in WT but not in DKO cells without detectable PP-IPs. This demonstrates a physiological role for PP-IPs in promoting cellular phosphate export by XPR1, as we demonstrated that IP_7_ binds to XPR1's SPX domain. Several aspects of mammalian cellular phosphate homeostasis are therefore regulated by PP-IPs. However, as XPR1 is our sole SPX domain-containing protein, the other phosphate-related phenotypes must be regulated by PP-IPs through other as yet unknown mechanisms and effectors.

Although the main IP6K product is 5-IP_7_, the DKO cells are devoid of all PP-IPs; the described effects on phosphate homeostasis could be due to the absence of 5-IP_7_, PP-IP_4_, or IP_8_. Knockout of the PPIP5Ks, the other class of enzyme responsible for PP-IPs synthesis, generates cells without IP_8_ but in which IP_7_ accumulates (30). *PPIP5K*^−/−^ cells have reduced cell proliferation and altered mitochondrial metabolism. These phenotypes can perhaps be attributed to the nonphysiological increase in IP_7_, because our generated DKO cells, lacking both IP_7_ and IP_8_, do not show any mitochondrial or proliferative defects. So far *PPIP5K*^−/−^ cells have not been characterized regarding any aspect of phosphate homeostasis: it will be interesting to investigate phosphate flux and its cellular accumulation in these cells, to verify if IP_8_ plays a role in these processes.

By removing PPIP5Ks' preferred substrate 5-IP_7_, the generated DKO cells are an ideal model in which to study PPIP5K activity on IP_6_. The resulting 1-IP_7_ isomer, representing less than 2% of cellular IP_7_ (25), is difficult to detect and thus to study. However, the appearance of detectable 1-IP_7_ in DKO cells treated with sodium fluoride to block PP-IPs recycling (24) demonstrates the presence of active PPIP5K enzymes, and should prompt further experiments to study their regulation and thus the synthesis and role of 1-IP_7_. Analysis of other inositol phosphates remains complex. The accumulation of the minor IP_5_ isomer I(1,2,4,5,6)P_5_ or I(2,3,4,5,6)P_5_, or both, in the DKO cells suggests that the proposed main route for I(1,2,4,5,6)P_5_ synthesis, IP6Ks dephosphorylating IP_6_ when AEC is low (9), does not occur *in vivo*. An unknown alternative pathway of I(1,2,4,5,6)P_5_ synthesis may therefore exist, which is nevertheless regulated by the IP6Ks. Perhaps in DKO cells, instead of phosphorylation by IP6Ks, IP_6_ enters the endolysosome system and is degraded by the phosphatase MINPP1 to I(1,2,4,5,6)P_5_ (31). Alternatively, IP6Ks have also been shown *in vitro* to synthesize IP_6_ from I(2,3,4,5,6)P_5_ under high AEC (9). If this occurs *in vivo*, then loss of IP6Ks could result in accumulation of this IP_5_ isomer, synthesized by an unknown route.

The latter suggestion could also explain why IP_6_ does not accumulate in DKO cells, despite the high IP_7_ and IP_8_ levels in WT HCT116 (23). The majority of IP_6_ is thought to be synthesized from I(1,3,4,5,6)P_5_ by the inositol pentakisphosphate 2-kinase (also known as IP5-2K, IPPK or IPK1) (32). If some of IP_6_ is actually generated from I(2,3,4,5,6)P_5_ by IP6Ks, the decrease in IP_7_ in DKO cells may not translate to IP_6_ increase, because there would be a concomitant reduction of IP_6_ synthesis. It is also possible that cellular IP_6_ levels are carefully homeostatically regulated. The study of flux between specific inositol phosphates, and post-translational regulation of inositol phosphate kinases, will be required to fully understand inositol metabolism.

The majority of characterization of the inositol phosphate metabolic pathways has been performed in the genetically tractable but simple *S. cerevisiae* yeast. CRISPR genome editing in mammalian cell lines allows deeper analysis of the function of specific inositol kinases or particular inositol phosphates or PP-IPs. Our IP6K DKO cells, as well as the *PPIP5K*^−/−^ cells (30), will be instrumental to fully appreciate the roles of these kinases and the PP-IPs made by them in mammalian physiology. Furthermore, generation of knocked-in endogenously kinase-dead cell lines will help us to appreciate nonenzymatic functions. Existing data rely on overexpression studies that could have artificial dominant-negative effects. Similarly, genomic tagging of the IP6Ks and PPIP5Ks will facilitate studies of their post-translational regulation, a subject of which almost nothing is currently known. Importantly, endogenous tagging will also allow physiological definition of their cellular localization, currently extrapolated from GFP fusion overexpression localization studies.

Knowing IP6Ks and PPIP5Ks intracellular localizations will be essential to fully understand their physiological roles. Our binding experiment revealed similar affinities for both IP_6_ and IP_7_ as SPX^XPR1^ ligands. In the WT cells, IP_6_ was 10 times more abundant than IP_7_ (calculated from data shown in Fig. 2), thus the crude binding data implies IP_6_ would be the prevailing ligand. However, specific IP6Ks localization could create a higher localized IP_7_ concentration, explaining the specific effect of IP_7_ on cellular phosphate export through XPR1. The situation here is similar to what has been reported for the yeast polyP-synthesizing VTC complex: binding studies gave similar affinities for IP_6_ and IP_7_ (4), but genetic work unquestionably indicates IP_7_ as the physiological VTC complex-regulating ligand (6). This functional selectivity could rely on hydrolysis of the high-energy pyrophosphate bond, whose presence distinguishes IP_7_ from IP_6_, perhaps inducing conformational changes in the bound protein or protein complex partner. NMR-based structural studies would be useful to test this hypothesis.

Structural studies have already influenced inositol phosphate experimental thinking. Over the past decade, several NMR and crystal structures have found an association of IP_6_ with proteins (33–37). These studies did not define a consensus binding pocket for IP_6_, suggesting that IP_6_–protein interactions reflect more the highly charged nature of the ligand than its stereochemical orientation. Its negative charge implies that IP_6_ would normally associate with bivalent cations, specifically magnesium, as has been demonstrated *in vitro* (38). However, none of the resolved IP_6_–protein structures, including IP_6_ with SPX domains, have shown IP_6_ coordinating magnesium or other cation. Notably, inositol phosphates have been found in crystals of proteins purified from eukaryotic cells (mammalian, insect, and yeast), without addition of exogenous inositol phosphates (33, 35, 39); these endogenously-derived ligands were also not associated with cations. Following these data, almost all the recent literature that includes inositol phosphate-binding experiments, including this study, uses magnesium-free buffers (4, 34, 35, 40). The issue remains contentious, as rationally IP_6_ should associate with magnesium. The recorded absence of cations suggests that highly phosphorylated inositol phosphates might exist in cation-free forms in cells, or that the cations are stripped off during protein binding. Because intracellular pH is emerging as a tightly controlled signaling mechanism (41, 42), it is intriguing to speculate on potential pH regulation of IP_6_-cation association: changes in cytosolic pH could free IP_6_ from magnesium, allowing and thus regulating its binding to protein effectors.

Phosphate uptake, as well as export, was reduced in the DKO cells. This is consistent with what was observed in *kcs1*Δ yeast, and with the initial PiUS characterization of IP6K2 (18). The regulation of phosphate flux in and out of cells must be fine-tuned to cellular phosphate needs. Therefore, phosphate-sensing mechanisms able to regulate these processes should exist in all cell types. It should be noted that the mammalian intracellular phosphate-sensing mechanisms are not yet known, although Pit1 is thought to sense extracellular concentrations (1). It is possible that IP_7_ is involved in this phosphate-sensing process, if not directly then at least indirectly, due to the change in phosphate level induced by IP_7_ depletion. DKO cells have access to a vast amount of more phosphate than the WT: in addition to the 1.3-fold higher free phosphate concentration, they possess more molecular sequestered phosphate in the form of ATP. Cytosolic ATP concentrations in unchallenged cells are around 2–4 mm (27). This represents quite a large pool of molecular sequestered phosphate. Therefore, small fluctuations in ATP or adenylate pools could well buffer mammalian cellular free phosphate needs. Cellular energy is sensed by the AMPK complex, which broadly responds to the relative amounts of AMP and ADP compared with ATP (28). During energy stress, active AMPK down-regulates anabolic ATP-consuming processes such as macromolecule synthesis, while up-regulating recycling pathways such as autophagy. The activity of AMPK, denoted by the α subunit's phosphorylation at Thr^172^, was decreased in DKO cells (Fig. 3*F*). This might suggest that IP_7_ signals phosphate availability through this complex. Further investigation into nutrient and energy signaling pathways in the DKO cells may be instructive, and are underway.

The designation of PP-IP_7_ as metabolic messenger also reflects a biochemical characteristic of the IP6K enzymes: they have a high *K_m_* for ATP, ∼1.4 mm (17, 43). As this is close to the physiological range for intracellular ATP, the level of IP_7_ in cells could be coupled to that of ATP, such that low ATP results in low IP_7_. This fits precisely with our finding that PP-IPs is required for phosphate export across the plasma membrane. It is likely that down-regulation of this export is desirable during periods of metabolic stress. In yeast, intracellular phosphate availability is sensed by the cyclin-dependent kinase inhibitor Pho81, in complex with the cyclin Pho80 and the cyclin-dependent kinase Pho85. Low phosphate conditions cause a conformational change in Pho81 that inhibits the kinase activity of Pho85, enabling the transcription factor Pho4 to up-regulate phosphate starvation genes. This conformational change in Pho81 depends on 1-IP_7_ (44). Mammalian genomes have no homologs of these Pho proteins. However, the ability of IP_7_ to control different aspects of phosphate homeostasis, through different mechanisms, in yeast and mammalian systems, underlines a fundamental role for PP-IPs in the regulation of phosphate metabolism.

We have shown that PP-IPs regulate cellular phosphate homeostasis, but as we use cell line models, were unable to investigate any regulation at the organismal level. In future, work using mice should clarify the physiological roles of PP-IPs in organismal phosphate homeostasis. Knockout mice for the individual IP6Ks exist, but there are no reported anomalies in serum phosphate level, kidney function, or bone physiology. Our results stress that more than one IP6K isoform must be knocked out to truly deplete PP-IPs. Studies characterizing the single KO mice phenotypes are supportive of IP_7_ as organismal metabolic regulator. *Ip6k1*^−/−^ mice are smaller than their WT littermates, with less body fat, and are resistant to obesity on a high fat diet (45, 46). They also show insulin sensitivity, with reduced plasma insulin and glucose levels. The *Ip6k3*^−/−^ mouse has a similar but weaker phenotype: small lean animals that show reduced plasma glucose with age (21). However, the *Ip6k3*^−/−^ mouse is not resistant to obesity, and serum insulin and glucose levels of *Ip6k2*^−/−^ mice are normal (47). A double or triple *Ip6k* knockout will allow understanding of the full picture, especially regarding phosphate homeostasis and its role in general metabolism. For example, renal-specific knockout of XPR1 in mice causes hypophosphatemia rickets and Fanconi syndrome (48). It will be important to revisit this result, studying the renal physiology of an *Ip6ks* knockout mouse, now that we know that PP-IPs are regulators of XPR1 activity.

## Materials and methods

### Maintenance and manipulation of cell lines

All cells were grown in DMEM (Invitrogen) supplemented with 10% FBS (Sigma) and 4.5 g/liter of glucose in a humidified atmosphere with 5% CO_2_. For [^3^H]inositol labeling, inositol-free DMEM (MP Biomedicals) with 10% dialyzed FBS (Sigma) was used. To starve cells of phosphate (0 mm phosphate), DMEM without sodium phosphate (ThermoFisher) supplemented with 10% dialyzed FBS was used. The usual phosphate concentration of DMEM is 0.9 mm. Cells were washed twice in the relevant starvation medium before incubation.

For sodium fluoride treatment, cells were seeded into 15-cm dishes and treated 24 h post-seeding with 10 mm sodium fluoride (Sigma) for 1 h. Cells were harvested by trypsinization.

For plasmid transfection experiments, cells were seeded into 6-well plates. Lipofectamine 2000 (ThermoFisher) was used with 1 μg of DNA/well. For siRNA experiments, cells were seeded into 12-well plates and transfected with 27 pmol of siXPR1 or Negative Control #1 siRNA (Silencer, ThermoFisher) using Lipofectamine 2000. The sequence of siXPR1 used was: 5′-gcuugccgcuguauuuaaatt-3′ (20). After 24 h, cells were harvested for RT-qPCR or labeled with [^32^P_i_] for phosphate release assay.

To measure cell growth, an SRB assay was performed (49). Cells were seeded into 96-well plates. At each time point, cells were fixed in 10% TCA. Fixed plates were stained with 0.05% sulforhodamine B (Sigma) in 1% acetic acid before reading absorbance at 500 nm using a spectrophotometer.

### Generation of IP6K KO cell lines

The human colon carcinoma cell line HCT116 was used to make knockouts as it is pseudo-diploid, and has easily detectable amounts of IP_7_ (23). Guide sequences targeting exon 5 of *IP6K1/2* were designed using the Zhang lab online design tool and cloned into plasmid pX335-U6-Chimeric_BB-CBh-hSpCas9n(D10A) (50). This plasmid was a gift from Feng Zhang (Addgene plasmid number 42335; RRID:Addgene_42335). Sequences used were: IP6K1, 5′-GCATCTTCAGGTCCAACACGC-3′ and 5′-CGATGACGCGTCAGCTGAGA-3′; IP6K2, 5′-GCACCTCGTAGCGGGAAGTC-3′ and 5′-GTGTGTCCTTGACCTCAAGAT-3′. Two guide sequences/plasmids were required per gene as we used the Cas9n D10A nickase to reduce off-target mutations. Cells were cotransfected with a puromycin resistance plasmid to allow selection. Colonies were initially screened by PCR using Expand High Fidelity Plus polymerase (Roche Applied Science) for indels >10 bp. Positive clones were sequenced to confirm mutations in exon 5. Two separate rounds of cell line generation were performed, generating several independent IP6K1^−/−^, IP6K2^−/−^, and DKO clones. Data shown are from representative clones.

### Analysis of inositol phosphates and polyP

Analysis of inositol phosphates by PAGE was performed as previously described (51). Briefly, cells in 15-cm dishes were trypsinized 48 h post-seeding and extracted using 1 m perchloric acid (Sigma). Titanium dioxide beads (Titansphere TiO 5 μm; GL Sciences) were used to pulldown inositol phosphates and other phosphate-rich molecules from the extracts. These extracts, normalized to protein concentration, were resolved using 35% PAGE gels as previously described (52) and visualized by toluidine blue (Sigma) staining. Images were obtained using a Epson desktop scanner.

To analyze polyP, cells were prepared as above except that phenol extraction was performed (6). Cells were vortexed for 5 min in 250 μl of acidic phenol and 250 μl of LETS buffer (10 mm Tris, pH 8, 100 mm LiCl, 10 mm EDTA, 0.5% SDS). The aqueous fraction was extracted again with chloroform. The subsequent aqueous fraction was ethanol precipitated and nucleic acids/polyP resuspended in resuspension buffer (10 mm Tris, pH 7, 0.5 mm EDTA, 0.05% SDS). Samples were normalized to RNA concentration and resolved using 30% PAGE gels and 4′,6-diamidino-2-phenylindole or toluidine blue staining. Control polyP-positive cells used were *Dictyostelium discoideum* AX4 strain starved for 24 h in KK2 buffer (20 mm potassium phosphate buffer, pH 6.8). This social amoeba accumulates polyP during starvation-induced development (53). Synthetic polyP of average length P13 (Sigma) or P100 (a kind gift from Dr. Toshikazu Shiba, RegeneTiss Co., Japan) was used as ladder.

For HPLC analysis, cells were seeded into 6-well plates in inositol-free DMEM. Trace amounts (5 μCi/ml) of *myo*-[^3^H]inositol (PerkinElmer Life Sciences) were added and cells incubated for 5 days. For analysis of Myc-Siw14 activity, cells were transfected with empty vector, pCMV-Myc-Siw14 (humanized sequence), or pCMV-Myc-Siw14 C214S (pyrophosphatase dead) constructs (29) using Lipofectamine 2000 (ThermoFisher) 24 h before harvesting. To harvest, cells were washed once in cold PBS before addition of cold 1 m perchloric acid. Samples were collected into microcentrifuge tubes after 10 min incubation on ice. Neutralized extracts were subjected to SAX-HPLC as previously described (54). Results were normalized to radioactivity in the lipid fraction, obtained by incubating the post-extraction cells in 1 m NaOH and 0.1% Triton X-100 overnight. Inositol phosphate peaks were identified using the relevant standards. These were extracted from yeast or synthesized *in vitro* before HPLC purification and desalting as previously described (55) The [^3^H]IP_6_ and [^3^H]I(1,3,4,5,6)P_5_ standards were purified from [^3^H]inositol-labeled *kcs1*Δ and *ipk1*Δ*kcs1*Δ yeast, respectively. To prepare [^3^H]IP_7_ and [^3^H]5PP-IP_4_, [^3^H]IP_6_ and [^3^H]I(1,3,4,5,6)P_5_, respectively, were incubated with recombinant IP6K1 in the presence of an ATP-recycling system, whereas [^3^H]I(2,3,4,5,6)P_5_ was prepared by dephosphorylating [^3^H]IP_6_ using IP6K1 in the presence of ADP, as described (9).

### Phosphate flux analysis

Analysis of uptake and release of [^32^P_i_] by pulse labeling was modified from a published protocol (20). These experiments were performed in DMEM containing phosphate. Cells in 12- or 6-well plates were treated with 0.5 μCi/ml of [^32^P_i_] (PerkinElmer Life Sciences) for 20 min at 37 °C. For uptake analysis, cells were then washed and lysed in 1 ml of 1% Triton X-100 before scintillation counting. Data were normalized to protein concentration of unlabeled control cells. For release analysis, labeled cells were washed, then incubated in fresh medium without radioactivity at 37 °C for 30 min. Media and cells were collected and analyzed. Results are given as extracellular [^32^P_i_] over cellular [^32^P_i_] cpm.

### Nucleotides measurements

For nucleotide analysis, cells were grown in 6-cm dishes and harvested by washing with cold PBS, scraping, and immediate quenching in cold 1 m perchloric acid. Samples were neutralized and nucleotides were analyzed by HPLC as previously described (56), using a Zorbax Extend-C18 4,6 × 150-mm column and Zorbax guard column (Agilent Technologies). Peaks were identified and quantified by comparison to spectra of standards (Sigma) acquired at the same time.

### Western blotting

Cells were lysed in RIPA buffer (10 mm Tris-HCl, pH 7.4, 140 mm NaCl, 1% Triton X-100, 0.1% sodium deoxycholate, 0.1% SDS, 1 mm EDTA, 1 mm EGTA) supplemented with protease and phosphatase inhibitor cocktails (Sigma). Lysates were cleared by centrifugation at 18,000 rpm for 5 min at 4 °C, and protein concentrations were measured by DC Protein Assay (Bio-Rad). Lysates were resolved using NuPAGE 4–12% bis-tris gels (Life Technologies) and proteins were transferred to nitrocellulose membranes. Membranes were blocked for 1 h in 5% nonfat milk in TBS-T (10 mm Tris base, 140 mm NaCl, 0.05% Tween) then blotted for the following primary antibodies at 1:500–1:1000 overnight in 3% milk:IP6K1 (HPA040825; Sigma), IP6K2 (sc-10425), β-tubulin (sc-9104), actin (sc-1616; Santa Cruz), oxidative phosphorylation mixture (458199), AMPK (AHO1332; ThermoFisher), phospho-AMPK Thr^172^ (number 2535), LKB1 (number 3047; Cell Signaling). Secondary horseradish peroxidase-conjugated antibodies (Sigma) were diluted in 3% milk. Signal was detected using Luminata Crescendo Western Substrate (Merck Millipore) and Amersham Biosciences Hyperfilm (VWR) and a film developer.

### Quantitative reverse transcription PCR (RT-qPCR)

Total RNA was extracted from cells using RNeasy Plus kit (Qiagen), and cDNA were generated using SuperScript III with oligo(dT)_20_ primers (ThermoFisher). Quantitative PCR was performed using MESA Blue qPCR mix (Eurogentec) and thermal cycler (Eppendorf). Primers used were: IP6K1 F, 5′-GAGGAGAAAGCCAGCCTGT-3′, R, 5′-TTCTCAAGCAGGAGGAACTTG-3′; IP6K2 F, 5′-AGTCATTGGTGTGCGTGTGT-3′, R, 5′-ACCAGCAGGGAGCTTGAGTA-3′; IP6K3 F, 5′-AAGACACCAACGGAAACCAG-3′, R, 5′-AGATCCAGGACACAGGGATG-3′; PPIP5K1 F. 5′-AGAAATGAAGCAGAGTGGCCT-3′, R, 5′-AAACAAGAGCTCATCTCGGTG-3′; PPIP5K2 F, 5′-GCTCATGGCAACAGGTTGTATC-3′, R, 5′-TCCTGTAGGTTAGGTGCGCT-3′; XPR1 F, 5′-CTGCTTGGCTTCGCTTCATC-3′, R, 5′-TCCGAGTGACCTCGTTCTTTG-3′; Pit1/SLC20A1 F, 5′-ATCCTCCATAAGGCAGATCCAG-3′, R, 5′-AGGATGGTACCCCACAGAGG-3′; GAPDH F, 5′-GTCGGAGTCAACGGATTTGG-3′, R, 5′-TCTCGCTCCTGGAAGATGGT-3′; β-actin F, 5′-GCCAACCGCGAGAAGATGA-3′, R, 5′-CATCACGATGCCAGTGGTA-3′. All primers were obtained from IDT DNA.

### Malachite green assay for free phosphate

Cells were seeded into 10-cm dishes, and harvested by washing and scraping in cold DMEM without phosphate. After lysis (20 mm Tris, pH 8, 150 mm NaCl, 1 mm EDTA, 1 mm EGTA, 1% Triton X-100, 10 mm sodium fluoride, 1× phosphatase inhibitor mixture), half of the sample was taken for protein quantification and the rest precipitated on ice for 15 min with 0.1 m final perchloric acid. Samples were cleared by centrifugation; the equivalent of 200 μg of protein was brought to 250 μl with 0.3 m perchloric acid before addition of 250 μl Malachite green reagent (0.8 mm Malachite green, 8.3 mm sodium molybdate, 0.7 m HCl, 0.05% Triton X-100). Absorbance was read at 650 nm using a spectrophotometer and compared with a sodium phosphate standard curve.

### Respirometry

To assess mitochondrial activity, oxygen consumption in intact cells was measured using a high-resolution respirometer (Oroboros), with the kind help of Dr. Will Kotiadis and Prof. Michael Duchen (University College London). Cells were suspended in the 2-ml chamber at 1 × 10^6^ cells/ml and respiration was allowed to stabilize at steady-state for 10 min (routine). Oligomycin (2 μm) was used to inhibit ATP synthase (leak), followed by the protonophore uncoupler carbonyl cyanide *p-*trifluoromethoxyphenylhydrazone (FCCP; 0.8 μm) until the maximum respiratory rate was reached (maximum uncoupled). Finally, antimycin A (AntA; 1 μm) was used to inhibit Complex III, for measurement of background respiration.

### Confocal microscopy

Cells were seeded onto glass coverslips, and imaged live or fixed using 4% formaldehyde for 15 min. Images were acquired using an SPE confocal microscope (Leica) using ×63 oil immersion lens. MitoTracker Deep Red (ThermoFisher), FITC-phalloidin, and Hoechst 33342 (Sigma) were used as stains.

### Competition-binding assay

The SPX domain of human XPR1 (SPX^XPR1^) was cloned by PCR into a modified His tag expression vector pHisTrcA plasmid (Invitrogen) using the following primers: F, 5′-GCAGTCGACCATGAAGTTCGCCGAGCACCTCT-3′, R, 5′-GCAGCGGCCGCTCACTGAGCAGCTCCCAAAGGGGGG-3′. Purification of the recombinant His-SPX^XPR1^ protein was carried out using AKTA protein purification systems (GE Healthcare) with a HisTrap 1-ml column, following the manufacturers' instructions. Inositol phosphate binding was determined using the PEG precipitation assay (57, 58). Recombinant protein was incubated with [^3^H]IP_6_ for 5 min in binding buffer (20 mm Tris-HCl, pH 7.4, 150 mm NaCl, 1 mm DTT) at room temperature before adding the nonradioactive competitive ligand and incubation for a further 15 min. Competitive ligands used were I(1,3,4,5,6)P_5_ (Sichem), IP_6_ (Calbiochem), and 5-IP_7_ and 1-IP_7_ that were synthesized as previously described (59, 60). The binding parameters were calculated using GraphPad Prism (GraphPad).

### Densitometry and statistical analysis

Densitometry analysis was performed using FIJI (61). Statistical analysis was performed using GraphPad Prism (GraphPad Software), using *t* test or ANOVA with Tukey post test, as appropriate.

## Author contributions

M. S. W. data curation; M. S. W. formal analysis; M. S. W. investigation; M. S. W. visualization; M. S. W. methodology; M. S. W. writing-original draft; M. S. W. project administration; M. S. W. and A. S. writing-review and editing; H. J. J. resources; A. S. conceptualization; A. S. supervision; A. S. funding acquisition.

## Supplementary Material

Supporting Information

## Footnotes

2 The abbreviations used are:

polyP: polymer inorganic polyphosphate
Pi: inorganic phosphate
AEC: adenylate energy charge
IP_6_: inositol hexakisphosphate, phytic acid
IP_7_: diphosphoinositol pentakisphosphate
IP_8_: bisdiphosphoinositol tetrakisphosphate
PP-IPs: inositol pyrophosphates
PP-IP_4_: diphosphoinositol tetrakisphosphate
AMPK: AMP-activated kinase
SRB: sulforhodamine B
DMEM: Dulbecco's modified Eagle's medium
FBS: fetal bovine serum
qPCR: quantitative PCR
FCCP: carbonyl cyanide *p-*trifluoromethoxyphenylhydrazone
AntA: antimycin A
DKO: double knockout
ANOVA: analysis of variance.
