# Supplementary material for: The inositol hexakisphosphate kinases IP6K1 and -2 regulate human cellular phosphate homeostasis, including XPR1-mediated phosphate export
Source: J Biol Chem. 2019 Jun 11;294(30):11597–608. doi: 10.1074/jbc.RA119.007848 (PMC6663863; doi:10.1074/jbc.RA119.007848)
Supplement: Supporting Information [file supp_294_30_11597__index.html]

The inositol hexakisphosphate kinases IP6K1 and -2 regulate human cellular phosphate homeostasis, including XPR1-mediated phosphate export — Control of mammalian phosphate homeostasis by PP-IPs — The inositol hexakisphosphate kinases IP6K1 and -2 regulate human cellular phosphate homeostasis, including XPR1-mediated phosphate export — Control of mammalian phosphate homeostasis by PP-IPs — Supporting Information 

# The inositol hexakisphosphate kinases IP6K1 and -2 regulate human cellular phosphate homeostasis, including XPR1-mediated phosphate export

## Supporting Information

- Supporting Information - Supporting Figures, Figure Legends, and Supporting Table
