# Supplementary material for: The inositol hexakisphosphate kinases IP6K1 and -2 regulate human cellular phosphate homeostasis, including XPR1-mediated phosphate export
Source: J Biol Chem. 2019 Jun 11;294(30):11597–608. doi: 10.1074/jbc.RA119.007848 (PMC6663863; doi:10.1074/jbc.RA119.007848)

The inositol hexakisphosphate kinases IP6K1 and -2 regulate human cellular phosphate homeostasis, including XPR1-mediated phosphate export

Miranda S. Wilson[1], Henning J. Jessen[2], Adolfo Saiardi[1]*

From the [1]MRC Laboratory for Molecular Cell Biology, University College London, London, UK; [2]Institute of Organic Chemistry, Albert-Ludwigs-University Freiburg, Freiburg, Germany

Running title: Control of mammalian phosphate homeostasis by PP-IPs

*To whom correspondence should be addressed: Adolfo Saiardi, email: a.saiardi@ucl.ac.uk

Supporting Materials
Supporting Figure legends:

Supporting Fig. S1. Generation and characterization of IP6K KO cells. (**A**) RTq-PCR analysis of IP6K mRNA transcripts in a variety of human cell lines, normalized to β-actin. (**B**) Treatment with 10 mM sodium fluoride (NaF) for 1 hour, followed by perchloric acid extraction, titanium dioxide purification and 35% PAGE with Toluidine blue staining. Standards for 1-IP_7 and 5-IP_7 (1.5 nmol each) were spiked into extracts just prior to gel loading. Synthetic polyP used as a ladder. (**C**) RTq-PCR analysis of PPIP5K1 (left) and PPIP5K2 (right) mRNA transcripts in KO cells, normalized to β-actin. No significance was found using ANOVA. Data show mean ± SD from 3 experiments. Gel in (B) is representative of 3 experiments.

Supporting Fig. S2. Inability to detect polyP in HCT116 cell extracts. (**A**) Phenol extracts from untreated HCT116 WT and DKO, and *D. discoideum* AX4 cells starved for 24 hours in KK2 buffer, were resolved by 30% PAGE and visualized with DAPI. Samples were quantified by co-purified RNA; 50 µg RNA was loaded per lane. (**B**) Cells were incubated in 0 mM phosphate for 24 hours, then in 0.9 mM phosphate with 0.5 µCi/ml [^{32}Pi] for 24 hours. Phenol extracts from $3x10^6$ cells were resolved by 25% PAGE and visualized first by Toluidine blue staining and then autoradiography. High molecular weight molecules such as genomic DNA or proteins remained in the wells. Gels are representative of experiments performed twice for (B) and three times for (A). P100 and P13 = synthetic polyP of average length 100 and 13 phosphates.

Supporting Fig. S3. Depletion of PP-IPs in HeLa cells using Siw14 pyrophosphatase also alters nucleotide levels. (**A**) SAX-HPLC of [^{3}H]inositol labeled cells. Cells were labeled for 5 days in inositol-free DMEM and transfected 24 hours before harvesting with plasmids encoding WT or pyrophosphatase-dead (C214S) humanized Myc-Siw14. (**B**) HPLC analysis of adenine nucleotides from Myc-Siw14 transfected cells. To account for differences in protein concentration measurements, panel on right shows data relative to Myc-Siw14 C214S-transfected results. Data show mean ± SD from 3 experiments. * = $p<0.05$, ** = $p<0.01$, ANOVA with Tukey post-test. Data in (A) are representative of experiments performed twice.

Supporting Fig. S4. Phosphate transporters expression is unchanged in DKO cells. (**A**) RTq-PCR analysis of Pit1/SLC20A1 mRNA transcripts, normalized to β-actin. (**B**) Analysis of XPR1 mRNA transcripts, normalized to β-actin. Data show mean ± SD from 4 experiments. No significance was found using t test.

	pmol/µg protein			
	**WT**	**DKO**	**IP6K2$^{-/-}$**	**IP6K1$^{-/-}$**
**AMP**	0.65 ± 0.18	0.61 ± 0.25	0.59 ± 0.41	0.52 ± 0.37
**ADP**	2.50 ± 0.48	2.64 ± 0.79	2.41 ± 0.85	2.46 ± 0.89
**ATP**	7.16 ± 1.17	11.85 ± 3.27	9.28 ± 3.94	8.88 ± 1.91
**AEC**	0.82 ± 0.02	0.87 ± 0.03	0.86 ± 0.03	0.86 ± 0.04

Supporting Table S1. Knockout of IP6Ks alters levels of adenine nucleotides. These HPLC data are shown as bar charts in Fig 2D, E. AEC = adenylate energy charge [(ATP+0.5ADP)/(ATP+ADP+AMP)]. Data show mean ± SD from 6 experiments.

Supporting Figure 4

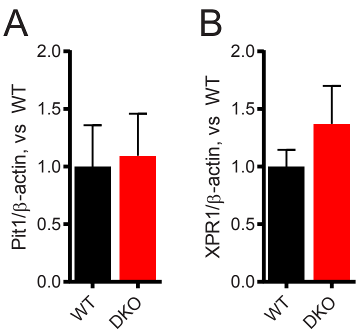

Supporting Figure 3

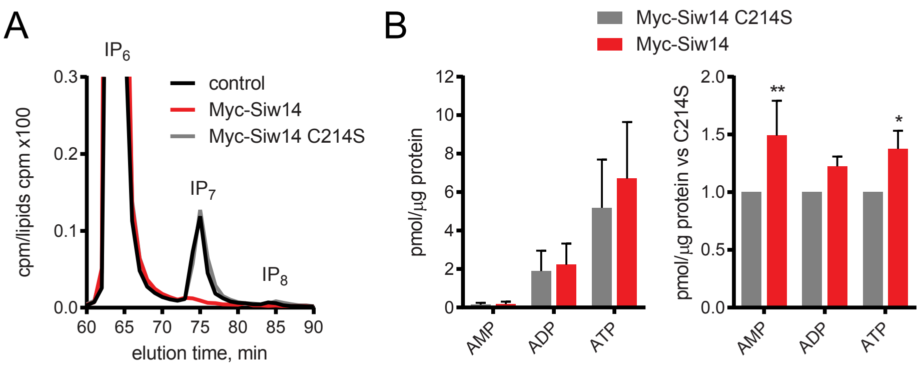

Supporting Figure 2

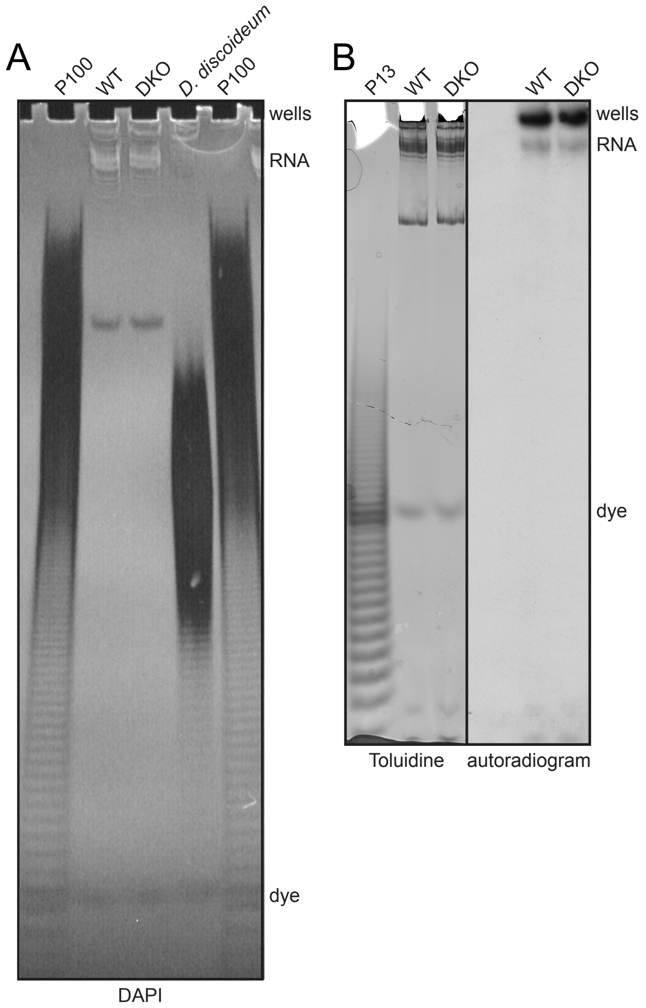

Supporting Figure 1

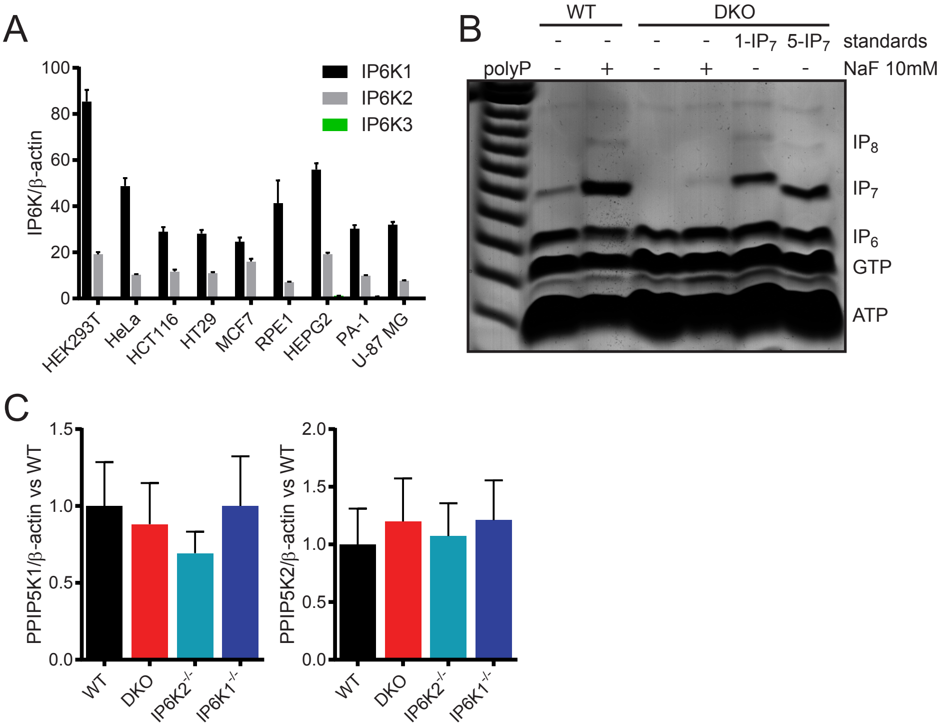

Supplement: Supporting Information [file supp_RA119.007848_143523_3_supp_342582_pskt0x.pdf]
